# Maternal and perinatal health research during emerging and ongoing epidemic threats: a landscape analysis and expert consultation

**DOI:** 10.1136/bmjgh-2023-014393

**Published:** 2024-03-07

**Authors:** Mercedes Bonet, Magdalena Babinska, Pierre Buekens, Shivaprasad S Goudar, Beate Kampmann, Marian Knight, Dana Meaney-Delman, Smaragda Lamprianou, Flor Muñoz Rivas, Andy Stergachis, Cristiana M Toscano, Joycelyn Bhatia, Sarah Chamberlain, Usman Chaudhry, Jacqueline Mills, Emily Serazin, Hannah Short, Asher Steene, Michael Wahlen, Olufemi T Oladapo

**Affiliations:** 1 UNDP/UNFPA/UNICEF/WHO/World Bank Special Programme of Research, Development and Research Training in Human Reproduction (HRP), Department of Sexual and Reproductive Health and Research, World Health Organization, Geneva, Switzerland; 2 Department of Epidemiology, Tulane School of Public Health and Tropical Medicine, New Orleans, Louisiana, USA; 3 Women's and Children's Health Research Unit, KLE Academy of Higher Education and Research's, Jawaharlal Nehru Medical College, Belgaum, Karnataka, India; 4 Charité Centre for Global Health, Universitätsmedizin Charité Berlin, Berlin, Germany; 5 National Perinatal Epidemiology Unit, Nuffield Department of Population Health, University of Oxford, Oxford, UK; 6 Division of Birth Defects and Infant Disorders, National Center on Birth Defects and Developmental Disabilities, Centers for Disease Control and Prevention, Atlanta, Georgia, USA; 7 Pharmacovigilance Team, Regulation and Prequalification Department, Access to Medicines and Health Products Division, World Health Organization, Geneva, Switzerland; 8 Departments of Pediatrics and Molecular Virology & Microbiology, Baylor College of Medicine, and Texas Children's Hospital, Houston, Texas, USA; 9 School of Pharmacy and School of Public Health, University of Washington, Seattle, Washington, USA; 10 Institute of Tropical Pathology and Public Health, Federal University of Goias, Goiania, Brazil; 11 Boston Consulting Group, London, UK

**Keywords:** Epidemiology, Maternal health, Obstetrics, Review, Infections, diseases, disorders, injuries

## Abstract

**Introduction:**

Pregnant women and their offspring are often at increased direct and indirect risks of adverse outcomes during epidemics and pandemics. A coordinated research response is paramount to ensure that this group is offered at least the same level of disease prevention, diagnosis, and care as the general population. We conducted a landscape analysis and held expert consultations to identify research efforts relevant to pregnant women affected by disease outbreaks, highlight gaps and challenges, and propose solutions to addressing them in a coordinated manner.

**Methods:**

Literature searches were conducted from 1 January 2015 to 22 March 2022 using Web of Science, Google Scholar and PubMed augmented by key informant interviews. Findings were reviewed and Quid analysis was performed to identify clusters and connectors across research networks followed by two expert consultations. These formed the basis for the development of an operational framework for maternal and perinatal research during epidemics.

**Results:**

Ninety-four relevant research efforts were identified. Although well suited to generating epidemiological data, the entire infrastructure to support a robust research response remains insufficient, particularly for use of medical products in pregnancy. Limitations in global governance, coordination, funding and data-gathering systems have slowed down research responses.

**Conclusion:**

Leveraging current research efforts while engaging multinational and regional networks may be the most effective way to scale up maternal and perinatal research preparedness and response. The findings of this landscape analysis and proposed operational framework will pave the way for developing a roadmap to guide coordination efforts, facilitate collaboration and ultimately promote rapid access to countermeasures and clinical care for pregnant women and their offspring in future epidemics.

**WHAT IS ALREADY KNOWN ON THIS TOPIC**

- Previous epidemics and pandemics highlighted the dearth of preparedness and response for maternal and perinatal health, resulting in delayed access to countermeasures for pregnant women and their offspring, despite them often being identified as a group at increased risk of severe disease outcomes.
- Existing literature evaluates gaps in approaches for alleviating gender inequality in future public health emergencies and the impacts of the COVID-19 pandemic on maternal and perinatal health services

**WHAT THIS STUDY ADDS**

- This study provides a comprehensive overview of existing research efforts and key areas of focus relevant to maternal and perinatal health, identifying current gaps and exposing shortcomings in existing infrastructure. It proposes an operational framework for improving conduct of maternal and perinatal heath research in the context of emerging and ongoing epidemic threats.

**HOW THIS STUDY MIGHT AFFECT RESEARCH, PRACTICE OR POLICY**

- The findings of this landscape analysis and proposed operational framework will pave the way for developing a roadmap to guide coordination efforts, facilitate collaboration and ultimately promote rapid access to countermeasures and clinical care for pregnant women and their offspring in future epidemics.

## Introduction

The likelihood of infectious disease outbreaks, epidemics and pandemics is increasing and is expected to triple over the coming decades,^1^ due to a number of contributing factors such as increased travel, urbanisation and climate change.^2^ Historically, the emergence of epidemic-prone diseases, including Ebola, Zika and respiratory infections such as severe acute respiratory syndrome (SARS), Middle East respiratory syndrome (MERS) and influenza A/H5N1 and A/H1N1 has caused global panic and alarm. However, disease emergence has often been followed by underinvestment in capacity strengthening, integrated surveillance and protection of populations during the recovery phase.^3^ In this context, the ability to quickly gather information on the natural course of disease progression, clinical characteristics and pathophysiology is necessary for the development of prevention and clinical care strategies and guidelines, as well as for the planning, design and delivery of care.^3^

Preparedness is key to reducing the impact of future disease outbreaks, and there are a number of lessons to be learnt from the experience of the COVID-19 pandemic, where prepandemic response planning was limited, and handling of the health emergency at the global level was a considerable challenge.^4^ In the aftermath of the pandemic, the international community has called for strengthening of health emergency preparedness, response and resilience architecture^1^ to better understand the distribution of priority emerging infectious diseases, together with drivers of transmission, natural history, clinical characteristics, and disease pathophysiology. This can help guide preparedness planning and strengthen health systems to ensure that they can effectively anticipate, respond to and recover from the impacts of any health emergencies.^3^ Integral to this response is the WHO Research and Development (R&D) Blueprint, which brings together key stakeholders to identify gaps and accelerate research for accurate diagnostic assays, novel therapeutics and effective vaccines against priority pathogens.^5–7^

It is now globally recognised that a comprehensive research response to emerging and ongoing epidemic threats can and should contribute to improve our understading of how these affect health and access to healthcare for women and children, in addition to their social and economic burden.^3^ Often, subpopulations, such as pregnant women and their offspring, are at higher risk both directly from the disease and from indirect factors. For example, pregnant women may be more likely to experience severe disease compared with non-pregnant women, as was noted during the COVID-19 and the 2009 influenza pandemics,^8 9^ or their offspring may be at increased risk for developmental abnormalities, such as the association between microcephaly and maternal Zika infection observed during the 2015 outbreak in Brazil.^10 11^ In addition to direct disease effects, pregnant women and their offspring are likely to be impacted by indirect effects, such as decreased access to maternity services, and increased childcare demands on working mothers during lockdown situations.^12 13^ Furthermore, pregnant women are generally excluded from clinical trials of medicines and vaccines, resulting in delayed access to potentially life-saving treatments or preventative interventions.^14–17^

Our objective was to evaluate the current maternal and perinatal research landscape and identify major gaps and challenges to delivering a coordinated and rapid research response to emerging and ongoing epidemic threats. We present the integrated findings of a landscape analysis, discussions with key informants (KIs) and outcomes of two expert consultations. We also propose an operational framework for maternal and perinatal research to be applied during ongoing and emerging epidemic threats.

## Methods

This landscape and gap analysis involved compilation and description of current research efforts relevant to maternal and perinatal health during ongoing and emerging epidemic threats. As such, it formed the basis for a series of consultations to further identify main challenges and opportunities for coordination and generate ideas of how current research efforts could be leveraged to address gaps. These supported development of an operational framework for improved maternal and perinatal health research during epidemics and pandemics. A steering committee was established to oversee and provide technical guidance at various stages of the project.

### Landscape and gap analysis

#### Desk review: search strategies and selection criteria

Initial searches were performed on Web of Science from 1 January 2015 to 22 March 2022 using three search strings including population (eg, maternal/pregnancy), topic area (eg, COVID-19, other infections) and methodology (eg, various study designs) (see online supplemental material 1). Where the initial publications referenced other relevant publications, research networks or authors, Google Scholar and PubMed were examined (using the same key search terms) to ensure completeness of the searches. In parallel, a similar search was performed across grey literature, including governmental websites, relevant non-governmental and international organisations, conference proceedings, clinical trial registers, existing research effort websites and associated networks’ sites, as well as targeted Google searches.

10.1136/bmjgh-2023-014393.supp1 Supplementary data

A research ‘effort’ was defined as a persistent data generation or aggregation exercise, which could be an individual study or a network or collaboration. Search results were filtered to exclude efforts considered to be beyond the scope of the study (eg, only testing interventions in neonates) or focused on multiyear/lifelong longitudinal cohort studies’ or those that had otherwise been terminated. Broader efforts, such as the WHO Programme for International Drug Monitoring^18^ and ISARIC network,^19^ were also excluded.

Preliminary findings from the literature review, grey literature and interviews were filtered against these screening criteria through manual review. The retrieved articles were screened by title and abstract to single out relevant full-text documents to be evaluated against the inclusion criteria. A data extraction form was used to extract information on the characteristics of those efforts (see online supplemental material 2), as well as opportunities and challenges pertaining to maternal and perinatal health research during epidemics and pandemics. What remained at the end of the filtering process was included in the landscape analysis.

#### Preliminary contacts with KIs

KIs were selected among the members of WHO steering committee, principal investigators or network members of efforts identified through the literature search. In total, 23 experts were contacted to identify further research efforts, gather more information on efforts led by KIs and gain insights on opportunities and challenges for collaboration.

#### Quid analysis

Findings of the literature search described above were validated using Quid (Quid, Business Intelligence Software, http://quid.com), an artificial intelligence software. The results were cross-checked and tested via Quid analysis to address biases and cover blind spots. This analysis allowed for finding gaps in the research landscape and clustering authors and research focuses and topics (eg, Zika, birth and morbidity) to detect networks and key individuals linking efforts, and to identify disparate, poorly linked clusters for which further investigation and outreach might be needed.

#### Synthesis of findings

Associated study publications, protocols and websites were reviewed to determine the population scope (eg, maternal, neonatal, both or general population), the region where the effort was active, operational period (research duration), type of research focus (eg, observational, interventional, surveillance) and topic area (eg, morbidity, outbreak/epidemic). Results of desk research and expert interviews were used to evaluate research efforts and better understand the full scope of activities and related publications. When there was evidence of previous pandemic and epidemic-related work, emergency focus was included as part of the research scope. Furthermore, key networks of clusters and authors serving as connections were visually identified using Quid analysis. To gain additional understanding, a deeper characterisation of selected efforts (exemplars) across a range of geographies and types was conducted. Key themes emerging from interactions with KIs were also identified and used to inform subsequent technical consultations.

### Technical consultations

Two expert consultations were conducted in June 2022 and May 2023 to reflect on the results of the landscape analysis, learn from challenges and opportunities of exemplars, discuss an operational framework, and identify needs and next steps to produce concrete and actionable outputs for improved maternal and perinatal health research during epidemics and pandemics. A total of 33 attendees with broad expertise and relevant clinical and academic experience attended the meetings. Among them, 22 were women and 11 were men; 11 experts came from low-income and middle-income countries (LMICs) while the remaining 22 experts represented high-income countries (HICs), most with direct experience in coordinating or supporting research in Africa, Asia and Latin America. In terms of the geographical representation of the WHO regions, 3 people came from Africa, 15 from the Americas, 2 from Eastern Mediterranean, 10 from Europe, 2 from South-East Asia and 1 person from the Western Pacific.

## Results

Overall, literature searches identified 3023 unique articles which were reviewed to identify relevant efforts corresponding with agreed definitions. Some articles yielded multiple efforts, while others yielded none. At the end of this process, a total of 94 research efforts considered relevant for maternal and perinatal health research during future outbreaks were identified (see online supplemental material 3). The landscape analysis and expert consultations yielded three key findings leading to the development of an operational framework.

### Finding 1: substantial research efforts exist; there is sufficient infrastructure to support robust maternal and perinatal health research during outbreaks mainly in high-income settings

**Figure 1:**
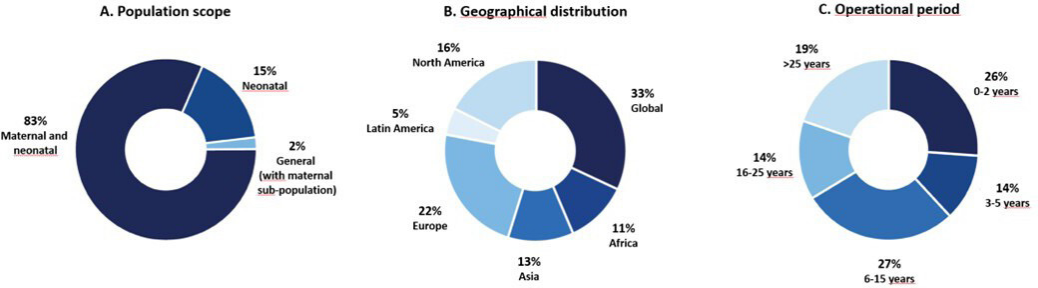
Characteristics of research efforts identified through literature searches and expert consultations.

Multiple relevant research efforts are already in place. In total, 83% (78/94) of research efforts focused predominantly on both maternal and neonatal health (figure 1A), with few efforts in the general population also including pregnant populations (2%, 2/94). These efforts have a broad geographical distribution, with 33% (31/94) being global efforts, 38% (36/94) originating from Europe or North America and 29% (27/94) originating from the rest of the world (figure 1B). Considerably fewer efforts were identified in Latin America, and there were no efforts solely based in the Eastern Mediterranean region. Data on duration were available for 81 research efforts, with the majority (60%) being operational for more than 5 years and 19% for more than 25 years (figure 1C). Many of these efforts had been successfully used during the COVID-19 pandemic by leveraging existing protocols and clinical trials to collect data on COVID-19 burden, pregnancy outcomes and use of medicines in pregnancy.^20–22^

Quid analysis showed that the over 3000 articles identified were authored by more than 20 000 researchers, in research networks consisting of more than 65 000 specific collaborations. Overall, the research ecosystem was predominantly comprised of discrete small clusters of research, with few connections (online supplemental figure 1a). In total, 31 clusters included 0–5 authors, 10 included 6–10 authors and 11 included >10 authors. The most connected networks contained several of the largest clusters (online supplemental figure 1b). The research landscape covered 16 major maternal and perinatal health topics, the most common being low-resource challenges (11%), diet and nutrition (11%) and vaccinations (12%; Online supplemental figure 1c).

Existing multicountry or regional networks may be the fastest path to improving maternal and perinatal health research during outbreaks. Large multicountry or regional networks already exist across epidemiology, research and development, postauthorization surveillance and advocacy. The International Network of Obstetric Survey Systems (INOSS) (https://www.npeu.ox.ac.uk/inoss), the Global Network for Women’s and Children’s Health Research (https://globalnetwork.azurewebsites.net/),^23^ HIV/AIDS Clinical Trials Units and Clinical Research Sites (https://www.niaid.nih.gov/research/hivaids-clinical-trials-units-and-clinical-research-sites) and NEOCOSUR (https://neocosur.uc.cl/neocosur/vista/index.php) are already coordinating research and enabling collaboration on randomised controlled trials and observational studies. Multisite networks increase access to larger and more diverse study populations, which in turn increases the generalisability of study findings. In addition, alignment and coordination within networks can allow prompt cascading of new studies, protocols or interventions to smaller satellite sites, which would not have been possible without cooperation within and among networks.

### Finding 2: existing infrastructure is best suited to provide epidemiological data; R&D including pregnant women during outbreaks is limited

Approximately 87% of the identified efforts are suited to support rapid generation of epidemiological data, 14% postauthorization surveillance data, whereas only 9% focus on research and development of interventions. Many efforts conducted activities that contributed towards multiple categories (eg, epidemiology and product development research).

Observational epidemiological efforts are suitable for rapidly leveraging the current infrastructure to describe the disease characteristics in outbreaks, epidemics and pandemics. Efforts such as the UK Obstetric Surveillance System (UKOSS),^20^ INOSS,^24^ the Global Network Maternal Newborn Health Registry,^23^ INTERCOVID^25^ and MA-Cov^26^ successfully adapted existing platforms during the COVID-19 pandemic. Still, certain barriers remain, such as the speed of ability to amend existing protocols. Relatively few efforts focused on development of interventions, and the majority centred on repurposing existing interventions rather than introducing novel ones. For example, excluding women from clinical trials resulted in a significant research gap during the COVID-19 pandemic,^14 15^ although there were efforts that advocated for improving inclusion of pregnant women in clinical trials (eg, ConcePTION^27^). Furthermore, significant barriers to inclusion of pregnant women in clinical trials persist for developers of medical products, ranging from perceived higher levels of legal liability and reputational damage to unknown risks to the pregnant woman and the fetus. At the same time, relatively few incentives are available, despite the existence of guidance supporting inclusion of pregnant women in clinical trials.^16 28 29^

### Finding 3: limitations in global governance, coordination and funding, and established data-gathering systems, cause delays in prompt, broad activation of research efforts during outbreaks

Establishing governance, coordination and funding plans at the time, rather than in advance, of emergencies such as the Zika virus disease outbreak and COVID-19 pandemic delayed generation of evidence critical to determining the burden of disease and guiding public health policies and clinical management. For example, most of the maternal and newborn health efforts during the Zika outbreak occurred after cases had peaked, therefore, missing critical periods for data collection and evidence generation for clinical decision-making. Efforts that required de novo development of studies and data-gathering systems, including protocols, ethics approvals, data sharing agreements, etc, responded more slowly than those that had these structures in place. Studies which leveraged existing protocols and systems (eg, UKOSS,^20^ Zika in Pregnancy in Honduras^30^ and INTERCOVID^25^ studies) during the COVID-19 pandemic resulted in more rapid generation of epidemiological data compared with those studies developed and launched after COVID-19 had already emerged. These existing research efforts would benefit from increased global coordination, including harmonisation of research protocols and preagreed data-sharing agreements and data analysis plans, to generate robust data that is applicable on an international scale. Specific funding to improve preparedness for research in pregnancy is not readily available, and many research efforts still struggle to obtain baseline funding. Funding for generating data concerning pregnant women is scarce and many research efforts are unable to secure and sustain baseline funding. It should be noted that prior to the COVID-19 pandemic, minimal investment was made for emergency preparedness and coordinated response, yet individual efforts (eg, vsafe,^31^ UKOSS^20^) received funding from emergency response.

### Operational framework for maternal and perinatal health research during emerging and ongoing epidemic threats

**Figure 2:**
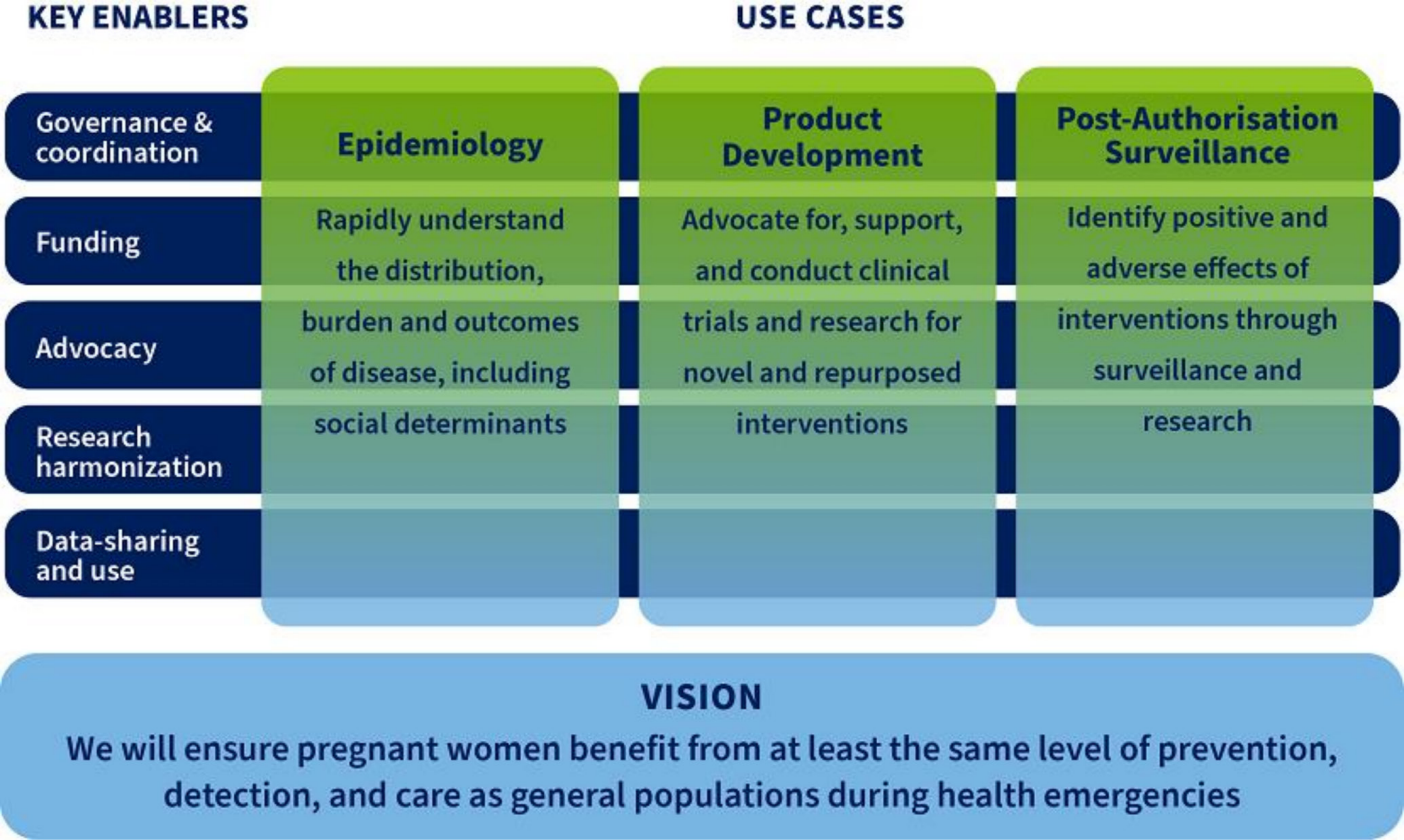
Operational framework for maternal and perinatal health research during emerging and ongoing epidemic threats.

The operational framework (figure 2) features three use cases (epidemiology, product development and postauthorisation surveillance) that address the key gaps identified in the landscape review and expert consultations. The ability to generate epidemiological data on distribution, risks and burden of disease, and to facilitate its use for informed response, clinical guidance and to enable prompt development of interventions is key. Conduct of trials that involve pregnant women where appropriate should support equitable development, access to and utilisation of interventions. Properly conducted postauthorisation surveillance activities would allow generation and communication of findings about the benefits and adverse effects of the use of medical products to further inform and update policy and practice.

The three use cases supported by five key enablers (governance and coordination, funding, advocacy, research harmonisation, data sharing and use) reflect interrelated actions that are needed to improve research and decision-making related to pregnancy during ongoing and existing epidemic threats. Good governance and enhanced coordination mechanism are necessary to enable, guide and oversee rapid research response encompassing research analyses and prompt dissemination of findings. Addressing health emergencies in a timely manner requires the presence of well-functioning sites and a pool of trained personnel. Researchers in maternal and perinatal health should work hand in hand with public health administrators, policy-makers and regulators on methods and data to be collected and shared in a manner that allows for informing policy and practice. The coordination mechanism should leverage existing platforms, ensuring that work is complimentary to and aligned with other preparedness initiatives directed at the general population. Establishing ‘centres of excellence’ or ‘sentinel sites’ should be supported as it would help close some of the existing gaps. Finally, opportunities ought to be created for research collaboration to continue at times when there are no outbreaks to maintain the existing infrastructure and promote continuous capacity building, particularly in low-resource settings.

In the initial phase, some funding would be required to establish major components of coordination and catalytic preparedness activities centred around capacity building, advocacy, harmonisation, and data sharing and use. Incremental funding would help maintain research readiness and research implementation during outbreaks, encompassing data collection, publication and dissemination, and translation of findings into policy and recommendations. There is a need to map funding opportunities and proactively engage with donors to promote preagreed funding priorities and mechanisms.

Building and maintaining relationships with key stakeholders to encourage continuous interest in involving pregnant populations in research would be an important enabler. Key stakeholders include researchers, health security and epidemiological surveillance actors, governments, policy-makers, industry, regulators, patient groups, and civil society representatives, among others. High-profile advocacy is needed to remove barriers to research concerning pregnant women. Collaboration with pharmaceutical companies, who are often disincentivised from involving pregnant women in clinical trials, is needed to better understand and address their concerns. Underlining the ethical aspects could substantially help in facilitating the inclusion of pregnant women in trials while encouraging the use of medical products in pregnancy, and disaggregation of epidemiological and surveillance data by pregnancy status. Another suggestion was to develop best practice guidance for community engagement and research, which would lead to meaningful engagement of women and civil society in epidemic and pandemic research. This covers efforts related to the dissemination of results and promotion of uptake of medicines and vaccines once those have been proven to be safe and effective. Advocating for the ‘general’ pandemic funding to include sexual and reproductive health funding is advisable as it would serve to ensure that other emergency preparedness efforts launched in the wake of COVID-19 pandemic consider pregnant women’s needs.

Finally, equitable approaches should be used for development and implementation of research and data sharing, and to obtain relevant ethics and regulatory approvals in a timely manner and using a risk-proportionate approach. Harmonisation of approaches, as opposed to complete standardisation across sites, is highly desirable. It would enable rapid research response, minimising delays to data collection, support rapid generation and synthesis of data, by addressing inconsistencies in outcome selection, measurement, and reporting. This entails development of harmonised research protocols for population-based epidemiological studies, clinical trials and postauthorisation surveillance based on an agreed set of core variables/outcomes and definitions, including patient-centred outcomes, preagreed global data sharing principles, authorship rules and publishing principles, in accordance with international regulations. A mapping and analysis of existing protocols, data analysis plans and data sharing agreements would inform development of standard procedures applicable across different countries and networks. This would serve to improve the availability of harmonised research tools and help streamline ethical review and approval processes while promoting data sharing and use by clinicians, regulatory authorities, policy-makers and others. Establishing fair agreements, including for sharing and using unpublished data, that consider the interests of countries and allow for research capacity building, while safeguarding those sharing data and study participants is crucial.

### Patient and public involvement

Patients were not involved in the design and conduct of the landscape analysis or expert consultations. Results of an ongoing systematic review on patient and public involvement in maternal and perinatal health research in LMICs were discussed at the expert consultation in June 2022.

## Discussion

This landscape analysis and consultative process identified 94 current research efforts applicable to maternal and perinatal research during emerging and ongoing epidemic threats. It further supported developing a better understanding of limitations and challenges to deliver a more coordinated and rapid research response on maternal and perinatal health during outbreaks. Many gaps were identified, ranging from clustered efforts towards epidemiological research to the need to scale up efforts related to R&D of medical products including pregnant women. In certain geographies, particularly in Latin America and Eastern Mediterranean, scarcity of research efforts was observed. Other regions suffered from lack of coordination, poor governance, insufficient funding and limited harmonisation of research and data sharing. An operational framework for improved maternal and perinatal health research has been proposed to address all those gaps. It spans across three ‘use cases’ (epidemiology, product development and postauthorisation surveillance) supported by five key enablers (governance and coordination, funding, advocacy, research harmonisation, and data sharing and use). The use cases would be ready for rapid deployment as per required geographical scope of an outbreak, thus allowing for a timelier decision making by policy-makers, health workers and pregnant women themselves.

While some global efforts covered all regions, the analysis revealed clustering of research towards certain regions and specific use cases. Yet, despite recent outbreaks of Zika, chikungunya and dengue relatively few research efforts were found in Latin America and that is concerning. Similarly, no efforts were identified in the Eastern Mediterranean where MERS first appeared. Going forward, a well-designed research infrastructure should be established and maintained in all regions to generate data as soon as the need arises. Although, our search strategy maximised identification of active research studies, networks and collaborations, from 2015, we may have missed some relevant research efforts. However, our findings showed that earlier research efforts, particularly those that emerged in response to respiratory diseases, were either discontinued or repurposed in the wake of the COVID-19 pandemic.

Ongoing efforts focused largely on collecting epidemiological data and relatively few efforts centred on product development in pregnant women. Global collaborative research networks that use harmonised protocols and simplified data collection systems have accelerated the process of evidence generation.^32^ Maintaining and expanding these research networks will help accelerate the response to future epidemics.

Epidemiological efforts will be vital for providing data on risks and outcomes during ongoing and emerging epidemic threats, and informing development of clinical trials and postauthorisation efforts that will encompass the population of pregnant women. Yet, additional engagement of stakeholders is desirable as it would help increase advocacy for appropriate inclusion of pregnant women in product development while allowing for a more rapid product delivery to this population in an emergency context. During the COVID-19 pandemic, a large number of clinical trials of selected vaccines and therapeutics systematically excluded pregnant women,^33^ while many of the products under evaluation had none or very low safety concerns during pregnancy.^14 15^ Barriers for inclusion of pregnant women in trials persist, despite continuous calls for generation of efficacy and safety data during pregnancy in the context of outbreaks.^16 33 34^ The lack of such clinical trial data hampers guideline development and public health advice. Ethical and regulatory frameworks and mechanisms defining when and how subpopulations such as pregnant women and children can and should be enrolled in clinical trials are needed to better address their needs. Currently, various guidance documents are being updated or developed at the international and national level, but none is specific to pregnancy research in the context of emerging and ongoing epidemic threats.

In contrast, effective networks and research studies are already underway or in place for conducting postauthorisation surveillance across many regions and they can be used during future outbreaks. Expanding them to cover additional geographies would provide a robust global picture of postauthorisation safety and allow for a rapid identification of any concerning signals in pregnant women or their offspring. The efforts potentially relevant to an emergency response identified in this landscape analysis fit into a broader landscape, which includes 8 maternal and neonatal data collection systems in LMICs,^35^ over 170 pharmacovigilance organisations globally,^18^ and 52 clinical trial networks focused specifically on infectious diseases, including in LMICs.^19^

Another gap that was identified referred to insufficient governance and lack of funding, leading to uncoordinated and slow research responses on maternal and perinatal health during epidemics and pandemics. Established sites, trained personnel and alignment among stakeholders are necessary for a coordinated emergency response which promotes inclusion of pregnant women in clinical trials, harmonises messaging and achieves a maximal impact within the resources available. Outside of epidemic and pandemic situations, the framework provides the potential to expand research focusing on pregnant women and their offspring at the global and regional level, allowing for an increased focus on other maternal and child health priorities. In addition, it serves to promote greater collaboration among research groups and institutions resulting in copublication of baseline data to be used by decision-makers as needed. Advocacy efforts underscore the importance of engaging pregnant women in research so that their needs are more likely to be considered in epidemic or pandemic situations. Stakeholder engagement is one of the key elements in achieving the vision of pregnant women benefiting from at least the same level of prevention, detection and care as the general population during epidemics and pandemics. This maximises preparedness to ensure that this group would not be left behind in the future.

In summary, this landscape analysis and associated consultations identified numerous gaps that should be addressed to improve generation of data on maternal and perinatal health, and inform timely decision-making by policy-makers, health workers and pregnant women themselves, particularly in LMIC settings. Having explored how existing maternal and perinatal health research platforms could be leveraged to address existing gaps and how they could be used to meet the need for a comprehensive global emergency response, it was determined that structures and mechanisms would need to be established to approach dealing with new epidemics or pandemics in a holistic and coherent manner. Using an operational framework based on three use-cases and five supporting key enablers, the WHO/Human Reproduction Programme aims to develop a roadmap to guide maternal and perinatal health research, facilitate data consolidation to enable faster decision-making and support readiness building. Efforts have already started and should be expanded for harmonisation of research protocols, and development of core outcomes to be collected for measuring maternal and perinatal health during future outbreaks.

## Data Availability

All data relevant to the study are included in the article or uploaded as online supplemental information.
